# Tirapazamine-loaded CalliSpheres microspheres: Preparation and characterization as a chemoembolization agent for liver cancer

**DOI:** 10.1016/j.mex.2023.102188

**Published:** 2023-04-16

**Authors:** Xin Zhang, Wei Hong, Qing Li, Yanyan Cao, Yiming Liu, Xiaopeng Guo, Lijie Zhang, Chuansheng Zheng, Bin Liang

**Affiliations:** Department of Radiology, Hubei Key Laboratory of Molecular Imaging, Union Hospital, Tongji Medical College, Huazhong University of Science and Technology, Wuhan, China

**Keywords:** Tirapazamine, Callispheres microspheres, Transcatheter arterial embolization, Pharmacokinetics, Methods for preparing and characterizing tirapazamine-loaded CalliSpheres microspheres

## Abstract

Tirapazamine (TPZ), a hypoxia-selective cytotoxic agent, has proved to exert synergistic tumor-killing activity with transcatheter arterial embolization (TAE) against liver cancer. This advances TPZ to transcatheter therapies for liver cancer, particularly in combination with drug-eluting microspheres. We describe methods for preparing and characterizing TPZ-loaded CalliSpheres microspheres (CSMTPZs) with regard to their properties as a chemoembolization agent, which includes 1) preparation of CSMTPZs and determination of drug loading level, 2) *in vitro* determination of TPZ release, 3) assessment of CSMTPZ size and appearance, and 4) determination of TPZ pharmacokinetics and intratumoral drug concentration *in vivo*. These methods can be adapted for further clinical I trial.

• This is to our knowledge the first methods for preparing and characterizing tirapazamine-loaded microspheres with regard to their properties as a chemoembolization agent
• Detailed protocols for preparation of CSMTPZs, determination of drug loading level, *in vitro* determination of TPZ release, assessment of CSMTPZ size and appearance, and *in vivo* determination of TPZ pharmacokinetics and intratumoral drug concentration
• Adaptable to experiments on other animal models and clinical trials

## Graphical abstract

**Figure:**
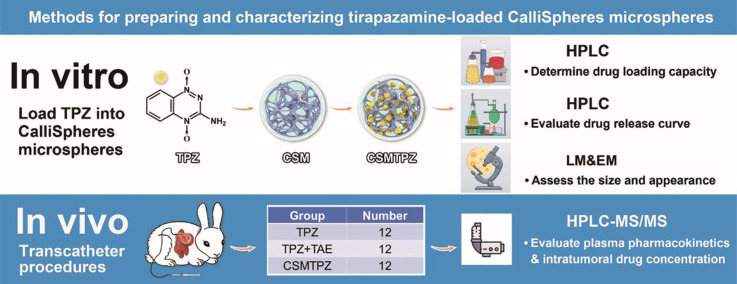

Specifications table

**Table:** 

Subject Area:	Interventional Radiology
More specific subject area:	Drug delivery and Pharmacokinetics
Method name:	Methods for preparing and characterizing tirapazamine-loaded CalliSpheres microspheres
Name and reference of original Method:	Liang B, Zhao D, Liu Y, Guo X, Zhang H, Zhang L, Zheng C. Chemoembolization of liver cancer with doxorubicin-loaded CalliSpheres microspheres: plasma pharmacokinetics, intratumoral drug concentration, and tumor necrosis in a rabbit model. Drug Deliv Transl Res, 2020 Feb;10(1):185–191.
Resource availability:	https://link.springer.com/article/10.1007/s13346–019–00672–9

## Background

Transcatheter arterial embolization (TAE), which induces ischemic/hypoxic tumor necrosis through blockage of the tumor-feeding artery, is a well-recognized therapy for unresectable hepatocellular carcinoma (HCC) [1]. However, the long-term efficacy of TAE remains unsatisfactory. The residual viable tumor after embolization may respond to hypoxia to survive and even progress [2,3]. Accordingly, the combination of TAE with hypoxia-selective cytotoxic agents probably constitutes an effective strategy to improve outcomes. Tirapazamine (TPZ), a representative hypoxia-selective cytotoxic agent [4], [5], has been evaluated in combination with conventional TAE. This combination therapy has demonstrated a promising synergistic antitumor effect against liver cancer [6], [7], [8], [9].

Drug-eluting microspheres have the ability to load chemotherapeutic agents and release them in a controlled mode. Transarterial chemoembolization (TACE) with the microspheres has shown more sustained and tumor-selective drug delivery as well as permanent embolization compared with conventional TACE [10]. We recently demonstrated that TPZ can be loaded into CalliSpheres microspheres as a chemoembolization agent targeting hypoxic tumor cells. In addition, the TPZ-loaded CalliSpheres microspheres (CSMTPZs) improved drug delivery to liver cancer, thus enhancing the synergy between TPZ and embolization against the tumor.

Herein, we describe the methods for preparing and characterizing the CSMTPZs in further detail.

### Overview of approach

• Load TPZ into CalliSpheres microspheres and determine loading capacity.
• Evaluate *in vitro* release of TPZ from CSMTPZs.
• Assess the size and appearance of the microspheres during TPZ loading and elution.
• Evaluate *in vivo* plasma pharmacokinetics and intratumoral TPZ concentration in an animal model.

Each of these steps is described in detail below, along with the results.

## Methods and results

### Basics

• Based on mechanical adsorption and ion interaction [11], TPZ was loaded into CalliSpheres microspheres.

### Preparation of CSMTPZs and determination of loading capacity

• CalliSpheres microspheres with the size of 100–300 µm (Jiangsu Hengrui Medicine Co., Ltd., Suzhou, China); TPZ (SR4233, ApexBio Technology Co., Ltd., Houston, TX, USA).
• TPZ was weighed and added into dimethyl sulfoxide, purified water, isotonic citrate buffer, and 7% HCl, respectively. TPZ-dimethyl sulfoxide solution of 20 mg/ml, TPZ-purified water solution of 1.5 mg/ml, TPZ-isotonic citrate buffer of 1.5 mg/ml, TPZ-7% HCl solution of 5 mg/ml, and TPZ-7% HCl solution of 10 mg/ml were prepared.
• CalliSpheres microspheres were weighed and added into vials. Each vial contained the equivalent of 1 g CalliSpheres microspheres. When the theoretical drug loading capacity was 35 mg TPZ/g microspheres, 1.75 ml TPZ-dimethyl sulfoxide solution, 23.3 ml TPZ- purified water solution, 23.3 ml TPZ-isotonic citrate buffer solution, 7 ml TPZ-7% HCl solution of 5 mg/ml, and 3.5 ml TPZ-7% HCl solution of 10 mg/ml were added into different vials, respectively. When the theoretical drug loading capacity was 70 mg TPZ/g microspheres, 3.5 ml TPZ-dimethyl sulfoxide solution, 46.6 ml TPZ-purified water solution, 46.6 ml TPZ-isotonic citrate buffer solution, 14 ml TPZ-7% HCl solution of 5 mg/ml, and 7 ml TPZ-7% HCl solution of 10 mg/ml were added into different vials, respectively.
• Shake the vials gently and start timing.
• An equal amount of supernatant was collected from mixtures at 15 min, 30 min, and 60 min.
• The content of residual TPZ in the supernatant was determined by high-performance liquid chromatographic (HPLC) analysis. Chromatographic separation was performed on a WATERS ACQUITY UPLC HSS T3 column (2.1 × 100 mm, 1.8 µm). The analytic conditions were as follows: injection volume, 20 µl; flow rate, 0.45 ml/min; column temperature, 35 °C. Solvent A was acetonitrile/ 0.1% H_3_PO_4_, Solvent B was water/ 0.1% H_3_PO_4_.
• The drug loading capacity at each time point was calculated.
• All experiments were performed in triplicate.

The results of the TPZ loading capacity with various drug loading doses, time-points and solutions are summarized in Table 1. The optimal loading capacity was 23.2 mg TPZ/g CSMs, when 70 mg of TPZ was dissolved into 7 ml of HCl 7% solution and then loaded 1 g of CSMs with the TPZ HCl solution for 60 min. We used the CSMTPZs with the optimal loading capacity for subsequent drug release experiments.

**Table 1:** TPZ loading capacity with various drug loading doses, time-points and solutions.

TIME (min)	DMSO		Purified water		Isotonic citrate buffer		7% HCl of 5 mg/ml		7% HCl of 10 mg/ml	
	35 mg/g	70 mg/g	35 mg/g	70 mg/g	35 mg/g	70 mg/g	35 mg/g	70 mg/g	35 mg/g	70 mg/g
15	10.1	12.0	3.0	5.1	3.3	6.1	9.8	16.1	15.3	22.3
30	10.6	12.6	3.7	6.2	3.9	7.2	9.4	15.9	15.3	22.3
60	11.6	12.6	3.9	6.8	4.2	7.8	9.9	15.9	15.3	23.2

TPZ, tirapazamine. DMSO, dimethyl sulfoxide.

### *In vitro* determination of TPZ release from CSMTPZs

• After achieving the optimal loading capacity, the supernatant of CSMTPZs solution was filtered and the surface of the microspheres was cleaned with fresh phosphate buffer solution (PBS, pH = 7.4, 10 mM).
• 0.2 g microspheres were weighed and added into a glass tube filled with 15 ml phosphate buffer solution (PBS, pH = 7.4, 10 mM). To promote the elution of TPZ from CSMTPZs, the glass tube was placed in a 200 rpm shaker at a constant temperature of 37 °C.
• An equal amount of release medium was collected at 0.5 h, 2 h, 4 h, 6 h, 8 h, 12 h, 24 h, 36 h, and 48 h. The same volume of fresh release medium was supplemented at each time point after collection.
• The content of TPZ in the release medium was also determined by high-performance liquid chromatographic (HPLC) analysis under the identical conditions as described above.
• The cumulative release rate at each time point was calculated.
• All experiments were performed in triplicate.

**Fig. 1:**
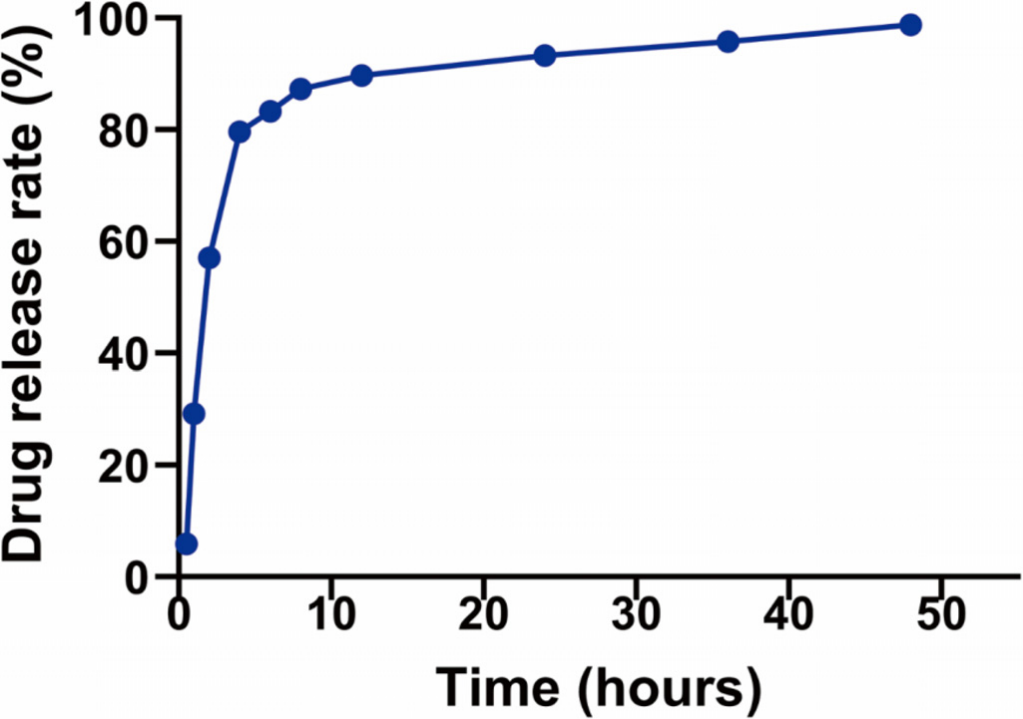
Release profiles of CSMTPZs with the loading of 23.2 mg TPZ/g CSMs.

The release profiles of CSMTPZs with the loading of 23.2 mg TPZ/g CSMs are shown in Fig. 1.

### Assessment of CSMTPZs size and appearance

• The color, size and shape of CSMTPZs during TPZ loading and release were evaluated under an aqueous solution by using an optical microscope (Motic, AE2000, China) with a SZSS-2000 camcorder (Motic, China). Microsphere sizing was measured with the SZSS-2000 image analysis software by taking two orthogonal measurements per microsphere, and the mean size was calculated by averaging 100 microspheres.
• The changes in surface profiles of the microspheres were evaluated by using a scanning electron microscope (Hitachi, SU8010, Japan). The microspheres were washed three times with ultra-pure water and then dripped onto the silicon wafer for drying. After being sputter-coated with gold, the microspheres were scanned by the scanning electron microscope.

**Fig. 2:**
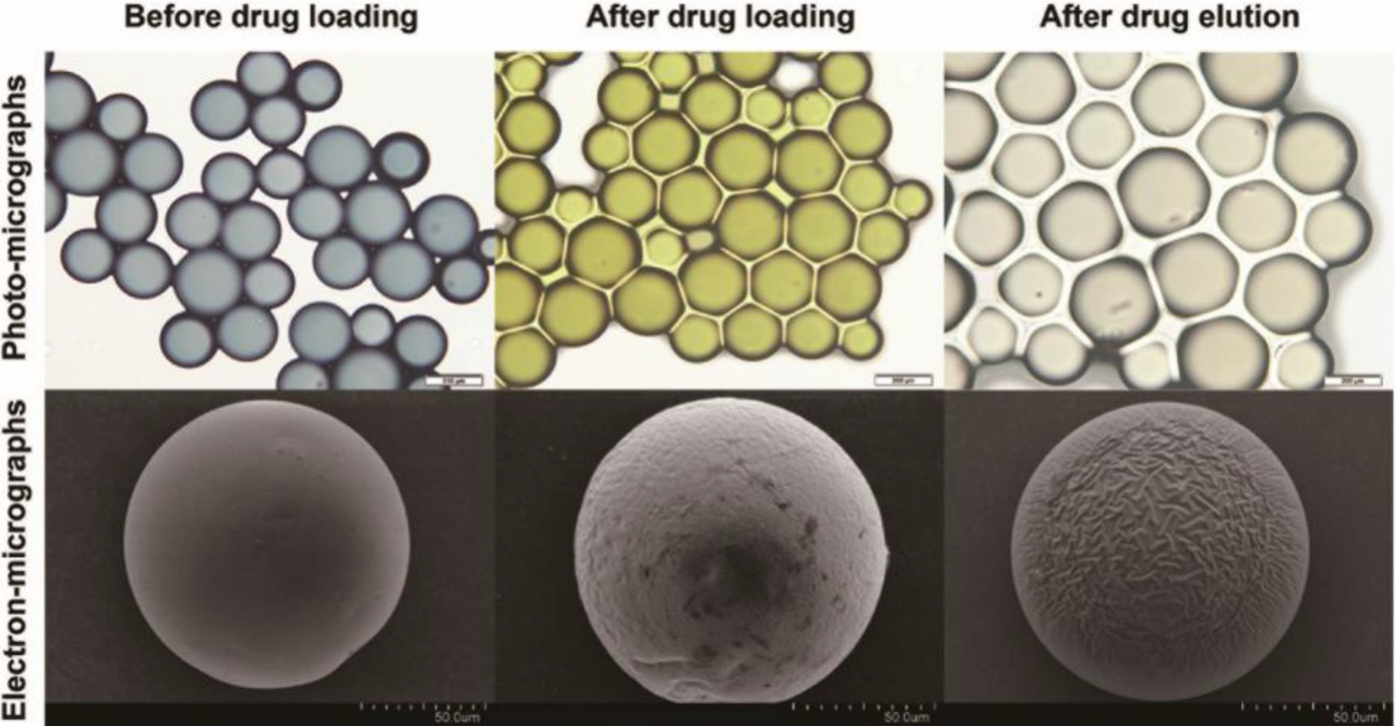
Photomicrographs of CSMTPZs during drug loading and release.

The color, size, shape and surface profiles of CSMTPZs during drug loading and release are shown in Fig. 2.

### Animal tumor model

• Thirty-six adult New Zealand white male rabbits weighing 2.0–2.5 kg were used to establish the liver tumor model. Animal experiments were performed in accordance with the Guide for the Care and Use of Laboratory Animals of Huazhong University of Science and Technology, as approved by the Animal Care Committee of Hubei Province, China.
• VX2 tumor cell suspension was injected into the right thigh muscle of a carrier rabbit. The tumor was harvested from the carrier rabbit and minced into 1–2 mm^3^ pieces under sterile conditions. These pieces were stored in saline for subsequent implantation.
• The animals were anesthetized with an intravenous injection of sodium pentobarbital (30 mg/kg body weight).
• After the preparation and sterilization of skin, an incision was made below the xiphoid process to expose the liver. A small wound with a depth of about 1 cm was made in the hepatic left lateral lobe. Then, a tumor piece and a gelatin sponge were implanted in sequence. The effect of the gelatin sponge was to prevent the tumor piece from falling out or liver bleeding. All rabbits were given intramuscular injections of penicillin after implantation.
• Two weeks after tumor implantation, each animal was scanned by MRI (Siemens, MAGNETOM Avanto, Germany) to assess the tumor growth situation.
• Interventional procedures were performed when the diameter of the tumor reached 1.0–1.5 cm.

### Groups

Thirty-six rabbits were randomly divided into three groups as follows:

⁎ TPZ group (*n* = 12): hepatic arterial infusion of TPZ;
⁎ TPZ + TAE group (*n* = 12): hepatic arterial infusion of TPZ followed by embolization;
⁎ CSMTPZ group (*n* = 12): hepatic arterial injection of CSMTPZs.

### Transcatheter procedures

• All rabbits were anesthetized in the same way as mentioned above. The femoral artery was separated after the preparation and sterilization of skin and the incision of skin and muscle.
• The femoral artery was punctured via modified Seldinger's technique.
• Transcatheter procedures were performed under the guidance of digital subtraction angiography (DSA, Siemens, Angiostar Plus, Germany). A 4-F visceral catheter (Cobra, Terumo, Japan) was used to determine the position of the hepatic artery at first. Further confirmation of the tumor and selection of the tumor feeding artery was performed using a 2.7-F coaxial microcatheter system (Progreat, Terumo). Subsequent treatments were carried out in the different groups.
• Rabbits in the TPZ group received an intraarterial injection of TPZ at a dose of 1 mg/kg body weight. Before injection, TPZ was pre-dissolved in 7% HCl solution, in a ratio of 10 mg to 1 ml. In the CSMTPZs group, we further loaded TPZ into CSMs and 23.2 mg TPZ/1 g CSMTPZs was achieved.
• Rabbits in the TPZ + TAE group received an intraarterial injection of TPZ at a dose of 1 mg/kg body weight, followed by blank microspheres (Jiangsu Hengrui Medicine Co. Ltd.). The arterial inflow occlusion was regarded as an embolization endpoint.
• Rabbits in the CSMTPZ group received an intraarterial injection of CSMTPZs at a TPZ dose equivalent to 1 mg/kg body weight, with or without additional embolization by blank microspheres.
• Finally, after removal of all sheaths and catheters, the femoral artery was ligated and the incision was closed.

### Sample collection

• Plasma samples were collected through marginal ear vein shortly before treatment and at 5 min, 10 min, 20 min, 40 min, 1 h, 4 h, 12 h, 24 h, 36 h, 48 h, and 72 h after treatment in each group. After centrifugation at 4000 rpm for 5 min, the upper serum was taken and stored at −80 °C for subsequent pharmacokinetic analysis.
• Tumor samples were collected at 1, 3, and 7 days after treatment in each group and stored at −80 °C for subsequent analysis of intratumor concentrations of TPZ and its main metabolites.

### Analysis of plasma pharmacokinetics and intratumoral drug concentration by HPLC-MS/MS

**Fig. 3:**
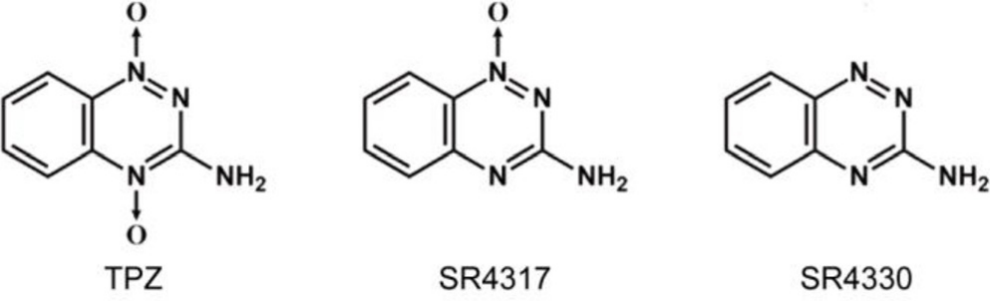
The structural formulas of TPZ and its main metabolites SR4317 and SR4330.
• Chromatographic separation was performed on a WATERS ACQUITY UPLC HSS T3 column (2.1 × 100 mm, 1.8 µm). The analytic conditions were as follows: injection volume, 5 µl; flow rate, 0.6 ml/min; column temperature, 40 °C. Solvent A was 0.1% formic acid/aqueous ammonium acetate (10 mM), Solvent B was 0.1% formic acid/ammonium acetate (10 mM) in methanol.
• For MS/MS, an AB Q-Trap 6500 (Applied Biosystems, USA) was used. The main parameters are as follows: positive electrospray ionization (ESI+); multiple reaction monitoring (MRM); spray voltage (IS), 3000 V; source temperature (TEM), 650 °C; GAS1, 65 psi; GAS2, 75 psi.
• The serum and homogenization of tumor tissue were analyzed.
• The data were acquired by Analyst1.6.3 software (Applied Biosystems, USA).

• The plasma pharmacokinetics and intratumoral drug concentration of TPZ and its main metabolites were analyzed by high-performance liquid chromatography with tandem mass spectrometric (HPLC-MS/MS) [12]. SR4317 and SR4330, the main metabolites of TPZ (SR4233), were obtained from the Department of Pharmacy, Tongji Medical College (Fig. 3).

**Fig. 4:**
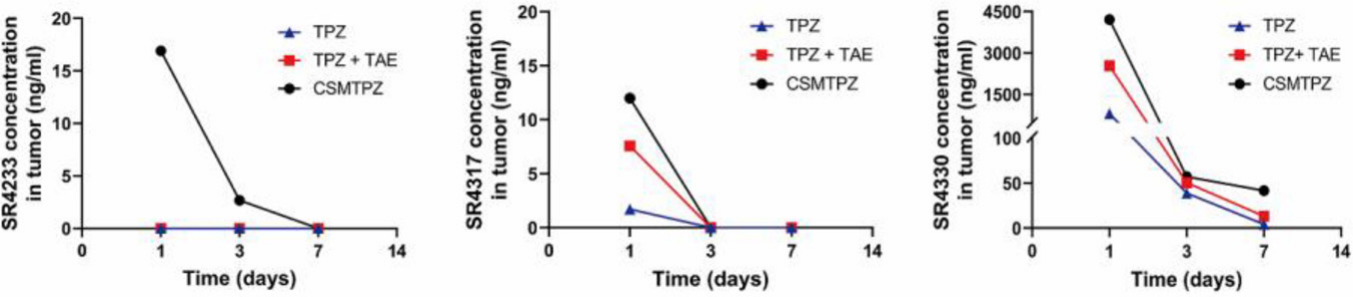
Intratumoral drug concentrations of TPZ and its metabolites at various timepoints after treatment in three groups.

The pharmacokinetic parameters and intratumoral drug concentrations of TPZ and its metabolites in three treatment groups are summarized in Table 2 and Fig. 4, respectively.

**Table 2:** Pharmacokinetic parameters TPZ and its metabolites in three treatment groups.

Drug	Group	AUC (0—∞)	T_1/2_ (h)	Cmax (µg/L)
SR4233	TPZ	176.723±78.099	0.237±0.038	412.78±182.044
	TPZ+TAE	123.934±39.727	0.398±0.198	199.9 ± 46.366
	CSMTPZ	40.263±13.435	0.286±0.102	61.672±14.695
SR4317	TPZ	115.212±36.352	0.753±0.498	112.728±37.843
	TPZ+TAE	103.234±81.876	0.965±0.475	73.178±42.231
	CSMTPZ	44.54±29.094	0.683±0.263	44.228±25.464
SR4330	TPZ	91.567±54.325	0.855±0.531	43.122±20.35
	TPZ+TAE	71.003±54.537	1.522±0.591	29.606±18.681
	CSMTPZ	39.495±28.703	0.973±0.985	29.046±6.065

TPZ, tirapazamine. TAE, transcatheter arterial embolization. CSMs, CalliSpheres Microspheres. CSMTPZ, TPZ-loaded CalliSpheres microsphere. AUC, area under the curve. T1/2, half-life. Cmax, maximum concentration.

## Conclusion

By using the aforementioned methods, we successfully prepared and characterized the CSMTPZS as a chemoembolization agent for liver cancer. These methods can be adapted for further clinical studies.

## Financial support

This work was supported by the 10.13039/501100001809National Natural Science Foundation of China [NSFC grant 81101134, 81771950 to B.L.] and China Health Promotion Foundation [CHPF grant XM_2018_011_0006_01 to B.L.].

## Declaration of Competing Interest

The authors declare that they have no known competing financial interests or personal relationships that could have appeared to influence the work reported in this paper.

## Data Availability

Data will be made available on request.
